# Association of Aspirin Use With Mortality Risk Among Older Adult Participants in the Prostate, Lung, Colorectal, and Ovarian Cancer Screening Trial

**DOI:** 10.1001/jamanetworkopen.2019.16729

**Published:** 2019-12-04

**Authors:** Holli A. Loomans-Kropp, Paul Pinsky, Yin Cao, Andrew T. Chan, Asad Umar

**Affiliations:** 1 Cancer Prevention Fellowship Program, Division of Cancer Prevention, National Cancer Institute, Rockville, Maryland; 2 Gastrointestinal and Other Cancers Branch, Division of Cancer Prevention, National Cancer Institute, Rockville, Maryland; 3 Early Detection Research Branch, Division of Cancer Prevention, National Cancer Institute, Rockville, Maryland; 4 Siteman Cancer Center, Division of Public Health Sciences, Department of Surgery, Washington University School of Medicine in St Louis, St Louis, Missouri; 5 Clinical and Translational Epidemiology Unit, Massachusetts General Hospital, Harvard Medical School, Boston; 6 Division of Gastroenterology, Massachusetts General Hospital, Boston; 7 Channing Division of Network Medicine, Department of Medicine, Brigham and Women’s Hospital, Harvard Medical School, Boston, Massachusetts; 8 Department of Immunology and Infectious Diseases, Harvard T.H. Chan School of Public Health, Boston, Massachusetts

## Abstract

**Importance:**

Aspirin use has been associated with reduced risk of cancer mortality, particularly of the colorectum. However, aspirin efficacy may be influenced by biological characteristics, such as obesity and age. With the increasing prevalence of obesity and conflicting data regarding the effect of aspirin in older adults, understanding the potential association of aspirin use with cancer mortality according to body mass index (BMI) and age is imperative.

**Objectives:**

To investigate the association of aspirin use with risk of all-cause, any cancer, gastrointestinal (GI) cancer, and colorectal cancer (CRC) mortality among older adults and to perform an exploratory analysis of the association of aspirin use with mortality stratified by BMI.

**Design, Setting, Participants:**

This cohort study evaluated aspirin use among participants aged 65 years and older in the Prostate, Lung, Colorectal, and Ovarian (PLCO) Cancer Screening Trial at baseline (November 8, 1993, to July 2, 2001) and follow-up (2006-2008). Analysis began in late 2018 and was completed in September 2019.

**Main Outcomes and Measures:**

All-cause, any cancer, GI cancer, or CRC mortality. Multivariable hazard ratios (HRs) and 95% CIs were calculated using time-varying Cox proportional hazards regression modeling, adjusting for additional factors.

**Results:**

A total of 146 152 individuals (mean [SD] age at baseline, 66.3 [2.4] years; 74 742 [51.1%] women; 129 446 [88.6%] non-Hispanic white) were included in analysis. The median (interquartile range) follow-up time was 12.5 (8.7-16.4) years, encompassing 1 822 164 person-years. Compared with no use, aspirin use 1 to 3 times per month was associated with reduced risk of all-cause mortality (HR, 0.84; 95% CI, 0.80-0.88; *P* < .001) and cancer mortality (HR, 0.87; 95% CI, 0.81-0.94; *P* < .001). Aspirin use 3 or more times per week was associated with decreased risk of mortality of all causes (HR, 0.81; 95% CI, 0.80-0.83; *P* < .001), any cancer (HR, 0.85; 95% CI, 0.81-0.88; *P* < .001), GI cancer (HR, 0.75; 95% CI, 0.66-0.84; *P* < .001), and CRC (HR, 0.71; 95% CI, 0.61-0.84; *P* < .001). When stratified by BMI (calculated as weight in kilograms divided by height in meters squared), aspirin use 3 or more times per week among individuals with BMI 20 to 24.9 was associated with reduced risk of all-cause mortality (HR, 0.82; 95% CI, 0.78-0.85; *P* < .001) and any cancer mortality (HR, 0.86; 95% CI, 0.79-0.82; *P* < .001). Among individuals with BMI 25 to 29.9, aspirin use 3 or more times per week was associated with reduced risk of all-cause mortality (HR, 0.82; 95% CI, 0.79-0.85; *P* < .001), any cancer mortality (HR, 0.86; 95% CI, 0.81-0.91; *P* < .001), GI cancer mortality (HR, 0.72; 95% CI, 0.60-0.86; *P* < .001), and CRC mortality (HR, 0.66; 95% CI, 0.51-0.85; *P* = .001).

**Conclusions and Relevance:**

In this cohort study, aspirin use 3 or more times per week was associated with a reduction in all-cause, cancer, GI cancer and CRC mortality in older adults.

## Key Points

**Question:**

Is aspirin use associated with reduced risk of mortality in older adults?

**Findings:**

This cohort study included 146 152 individuals from the Prostate, Lung, Colorectal, and Ovarian Cancer Screening Trial and found that aspirin use 3 or more times per week was associated with reduced risk of all-cause, cancer, gastrointestinal cancer, and colorectal cancer mortality.

**Meaning:**

These findings suggest that prophylactic aspirin use may reduce risk of mortality among older individuals.

## Teaser

This cohort study uses data from the Prostate, Lung, Colorectal, and Ovarian Cancer Screen Trial to examine the associations among regular aspirin use, obesity, and all-cause and cancer mortality among older adults.

## Introduction

Aspirin use of 10 years or more is estimated to reduce cancer incidence and mortality.^1,2,3,4^ Across epidemiological studies, the most significant reductions in risk have been noted in gastrointestinal (GI) cancers.^2,4^ Notably, results from the NIH-AARP study,^5^ the Nurses’ Health Study,^6^ and Health Professionals Follow-up Study^7,8^ demonstrated that long-term regular aspirin use was associated with reduced risk of obesity- and inflammation-associated cancers, particularly colorectal cancer (CRC). This result has been additionally illustrated in a 2010 meta-analysis of randomized clinical trials.^4^ The association of aspirin with reduced cancer incidence and mortality has been consistently robust among GI cancers; however, there is limited evidence suggesting that aspirin use is associated with reduced mortality risk among other cancer types.^3,9,10,11^ The US Preventive Services Task Force (USPSTF) recommends low-dose aspirin use for the prevention of cardiovascular disease and CRC among average-risk individuals aged 50 to 59 years with a 10% or greater 10-year risk of cardiovascular disease; however, individuals aged 60 to 69 years should have an individualized approach, while evidence for use among individuals 70 years and older remains insufficient.^12^ It is important to note that a 2015 systematic review of the literature by the USPSTF^11^ found that individuals with daily aspirin dosing up to 1200 mg for a duration of 4 or more years exhibited a significant reduction in all-cause and cancer mortality, demonstrating a similar benefit of low-dose and standard aspirin.

Recently, the results of the Aspirin in Reducing Events in the Elderly (ASPREE) study^13^ drew further attention of the potential cancer preventive effect of aspirin. The ASPREE study was a randomized clinical trial conducted in the United States and Australia investigating the efficacy of 100 mg of aspirin daily vs placebo in extending disability-free survival among elderly individuals (recruitment age 65 years or 70 years) during 4.7 years of follow-up.^13^ Unexpectedly, an increased risk of all-cause and cancer-related death was observed in the group randomized to aspirin.^14^

Thus, based on the results of the ASPREE randomized clinical trial that conflict with the preponderance of data showing a benefit of aspirin use, we examined the association of aspirin use with mortality risk among older adults enrolled in the Prostate, Lung, Colorectal, and Ovarian (PLCO) Cancer Screening Trial. In addition, given data suggesting that aspirin may be less effective among individuals who are overweight or obese, we conducted exploratory analyses stratified by body mass index (BMI), calculated as weight in kilograms divided by height in meters squared.^1^

## Methods

This study is a post hoc analysis of PLCO study data. The original trial was approved by the institutional review boards at all study sites (University of Alabama at Birmingham, Georgetown University, University of Pittsburgh, Washington University in St. Louis, University of Utah, University of Colorado, University of Minnesota, Pacific Health Research and Education Institute, Henry Ford Health System, and Marshfield Clinic Research Foundation). All enrolled participants provided written informed consent. The use of their data for additional studies and analysis was included in the original informed consent. This study adhered to the Strengthening the Reporting of Observational Studies in Epidemiology (STROBE) reporting guideline.

### Study Design

The design and methods of the PLCO Cancer Screening Trial have been previously described.^15,16^ Briefly, participants aged 55 to 74 years were randomized to the intervention (screening) or control arm at 10 screening centers in the United States between November 8, 1993, and July 2, 2001. Pertinent exclusion criteria for this study were individuals with a history of CRC or lung, prostate, or ovarian cancer, previous surgical removal of the colon, lung, or prostate, or, starting in 1995, individuals who had a colonoscopy, sigmoidoscopy, or barium enema in the 3 years prior to study randomization. This cohort included participants who were 65 years or older or survived until age 65, had a valid baseline questionnaire (BQ) after study enrollment, and had reported information on aspirin use at baseline. Completion of a follow-up supplemental questionnaire (SQ) by participants between 2006 and 2008 was not required for inclusion into the study cohort. All responses to the BQ and SQ were ascertained by self-report. The full content of both questionnaires has been published previously.^17,18^ Briefly, the BQ asked, “During the last 12 months, have you regularly used aspirin or aspirin-containing products, such as Bayer, Bufferin, or Anacin (Please do not include aspirin-free products such as Tylenol and Panadol).” If the respondent answered yes, the frequency categories were given as less than 2 per month, 2 to 3 per month, 1 per week, 2 per week, 3 to 4 per week, 1 per day, or 2 or more per day. The SQ asked, “During the last 12 months, about how often did you usually take aspirin (examples of aspirin include Bayer, Bufferin, Anacin, and baby aspirin)?” The frequency categories were given as none or less than once per month, 1 to 3 times per month, 1 to 2 times per week, 3 to 6 times per week, and 7 or more times per week. To allow for consistent values between BQs and SQs, we collapsed the aspirin use frequency variables into no aspirin use or less than once per month, 1 to 3 times per month, 1 to 2 times per week, and 3 or more times per week. Usual dose information was not available on the BQ; therefore, this analysis does not consider dose. We categorized BMI as less than 20, 20 to 24.9, 25 to 29.9, and 30 or higher.

Initial analysis of the PLCO Cancer Screening Trial was completed after 13 years of follow-up or on December 31, 2009, whichever came first.^15^ Deaths were ascertained via annual study update questionnaire, individual reports, or death certificates. Beginning in 2011, participants were reconsented for follow-up. This allowed for linkage to the National Death Index, which extended mortality follow-up for up to 20 years after randomization.^19^ For those reconsenting, the end of mortality follow-up was December 31, 2015 or their date of death, whichever came first. For the approximately 15% of individuals who were alive in 2011 but who declined extended follow-up, the end of mortality follow-up was their declining date, which was generally in 2011.

### Statistical Analysis

**Figure 1.**
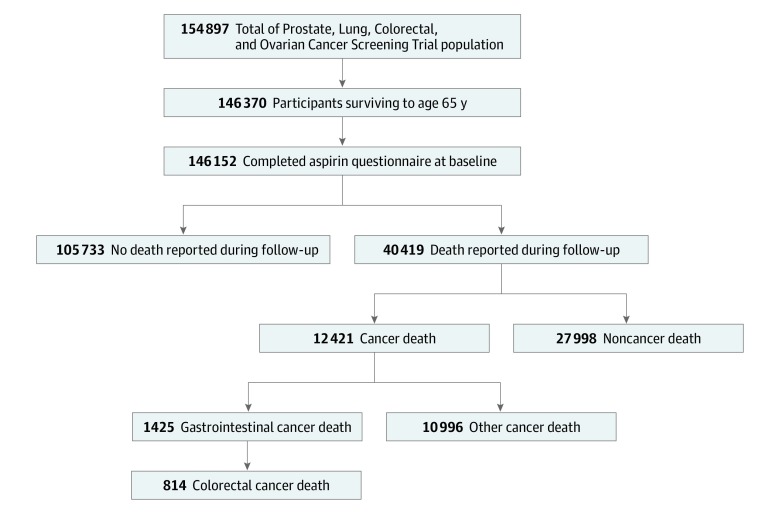
Flowchart of the Prostate, Lung, Colorectal, and Ovarian Cancer Screening Trial Cohort Used in the Study Analysis The current study cohort included participants who provided the baseline questionnaire with completed aspirin frequency and survived until age 65 years.

The purpose of this analysis was to assess the associations among aspirin use, BMI, and mortality of all causes, any cancer, gastrointestinal (GI) cancer, and CRC among individuals in the PLCO Cancer Screening Trial who survived to age 65 years and older (Figure 1). Participant follow-up time was measured from the time of cohort entry, which was either the date of PLCO Cancer Screening Trial enrollment or age 65 years, whichever occurred last, until the date of death or December 31, 2015, whichever came first. Survival time was measured from the time of cohort entry, until time of death of any cause. Hazard ratios (HRs) were calculated using Cox proportional hazards regression models to analyze the associations among aspirin use (no use, 1-3 times per month, 1-2 times per week, and ≥3 times per week), BMI (<20, 20-24.9, 25-29.9, ≥30), and mortality, controlling for covariates. As aspirin use, BMI, and other covariates may have changed between completion of the BQ and SQ, time-varying proportional hazards models were used. Initial aspirin use and covariate values were taken from the BQ for participants 65 years or older at randomization and for participants younger than 65 years at randomization who either did not fill out the SQ or filled it out after reaching age 65 years; initial values were taken from the SQ for those younger than 65 years at randomization completing the SQ before age 65 years. Among those with initial aspirin use and covariate values taken from the BQ and who also completed the SQ, values were updated at that time to the corresponding SQ values. Time-varying variables included in the model were aspirin use, BMI, smoking status (ie, never, current, or former smoker), history of myocardial infarction, history of stroke, history of hypertension, history of diabetes, and ibuprofen use 3 or more times per week. Static variables included in the models were sex (male or female), race/ethnicity (white non-Hispanic, black non-Hispanic, or other), and randomization arm (intervention or control). We additionally stratified by BMI to evaluate the association of aspirin use and BMI with mortality.

Finally, as an exploratory assessment of longitudinal patterns of aspirin use, we examined risk of mortality only among participants who had completed aspirin frequency questions on the BQ and SQ. Follow-up for this cohort subset began at age 65 years or completion of the SQ, whichever occurred last. Participants were categorized according to baseline and follow-up aspirin use as (1) consistent nonusers (use >1 time per week or no use at baseline and follow-up), (2) users at baseline only (use ≥1 time per week at baseline only), (3) users at follow-up only (use ≥1 time per week at follow-up only), or (4) consistent users (use ≥1 time per week at baseline and follow-up). We used weekly aspirin use as our metric in this analysis for consistency with previous analyses and to include the maximum number of participants with BQ and SQ data. Supplemental questionnaire variables included in the model were randomization arm, sex (male or female), race/ethnicity (white non-Hispanic, black non-Hispanic, or other), BMI, smoking status (never, current, or former smoker), history of myocardial infarction, history of stroke, history of hypertension, history of diabetes, and ibuprofen use 3 or more times per week.

Causes of death were determined by *International Classification of Diseases, Ninth Revision* (*ICD-9*) codes.^20^ All-cause mortality was defined as a reported death of any cause. Any cancer mortality was defined as a reported cause of death by cancer based on the official PLCO Cancer Screening Trial definitions, derived from standard *ICD-9* groupings. Cause of death by GI cancer was determined by *ICD-9* codes for esophagus (150), gastric (151), and colorectal (153 and 154) cancer, whereas CRC was determined by *ICD-9* codes for CRC only (153 and 154). All statistical analyses were performed in SAS statistical software version 9.4 (SAS Institute). *P* values were 2-tailed, and statistical significance was set at less than .05.

## Results

The eligibility criteria for the study analysis are outlined in Figure 1. After excluding individuals who died before age 65 years, there were 146 152 participants (mean [SD] age at baseline, 66.3 [2.4] years; 74 742 [51.1%] women; 129 446 [88.6%] non-Hispanic white) included in our analysis. The mean (SD) duration from baseline to completing the SQ was 9.1 (1.9) years and the median (range) was 9.2 (4.6-14.2) years, with a median (interquartile range) follow-up time of 12.5 8.7-16.4) years. Among the total cohort included in our study, 40 419 individuals (27.7%) died during follow-up, including 12 421 individuals (30.7%) who died of any cancer, 1425 individuals (3.5%) who died of GI cancers (including 353 individuals with esophageal cancer [2.8%] and 258 individuals with gastric cancer [2.1%]), and 814 individuals (6.6%) who died of CRC. Demographic characteristics of the cohort are summarized in Table 1.

**Table 1.** Characteristics of 146 152 Participants at Baseline

Characteristic	No. (%)					
	Total^a^^,^^b^	No Reported Death	Mortality			
			All-Cause	Cancer	GI Cancer	CRC
No.	146 152	105 728	40 419	12 421	1425	814
Age, y						
65^b^	100 046 (68.5)	81 933 (77.5)	18 113 (44.8)	6631 (53.4)	735 (51.6)	414 (50.9)
66-69	26 116 (17.9)	15 348 (14.5)	10 768 (26.6)	3138 (25.3)	359 (25.2)	209 (25.7)
70-74	19 981 (13.7)	8449 (8.0)	11 532 (28.5)	2651 (21.3)	330 (23.2)	190 (23.3)
≥75	9 (0.0)	3 (0.0)	6 (0.0)	1 (0.0)	1 (0.1)	1 (0.1)
Sex						
Men	71 410 (48.9)	47 090 (44.5)	24 320 (60.2)	7410 (59.7)	957 (67.2)	489 (60.1)
Women	74 742 (51.1)	58 643 (55.5)	16 099 (39.8)	5019 (40.3)	468 (32.8)	325 (39.9)
Race/ethnicity						
Non-Hispanic						
White	129 446 (88.6)	94 034 (88.9)	35 412 (87.6)	10 929 (88.0)	1202 (84.4)	690 (84.8)
Black	7285 (5.0)	4731 (4.5)	2554 (6.3)	765 (6.2)	109 (7.7)	66 (8.1)
Other^c^	9421 (6.5)	6968 (6.6)	2453 (6.1)	727 (5.9)	114 (8.0)	58 (7.1)
Randomization group						
Intervention	73 827 (50.5)	53 431 (50.5)	20 396 (50.5)	6262 (50.4)	646 (45.3)	349 (42.9)
Control	72 325 (49.5)	52 302 (49.5)	20 023 (49.5)	6159 (49.6)	779 (54.7)	465 (57.1)
Smoking status						
Never	68 103 (46.6)	53 162 (50.3)	14 941 (37.0)	4023 (32.4)	519 (36.5)	335 (41.2)
Current	150 022 (10.3)	8622 (8.2)	6400 (15.8)	2455 (19.8)	188 (13.2)	90 (11.1)
Former	63 007 (43.1)	43 938 (42.6)	19 069 (472)	5942 (47.8)	717 (50.4)	389 (47.8)
Body mass index^d^	3945 (2.7)	2603 (2.5)	1342 (3.3)	359 (2.9)	36 (2.6)	19 (2.3)
<20						
20-24.9	44 573 (30.5)	32 924 (31.1)	11 649 (28.8)	3646 (29.4)	383 (27.3)	222 (27.3)
25-29.9	61 114 (41.8)	44 481 (42.1)	16 633 (41.2)	5305 (42.7)	609 (43.4)	332 (40.8)
≥30	34 493 (23.6)	24 375 (23.1)	10 118 (25.0)	2926 (23.6)	374 (26.7)	225 (27.6)
Reported aspirin use in the last 12 mo						
None or <1/mo	74 908 (51.3)	55 701 (52.7)	19 207 (47.5)	6214 (50.0)	733 (51.4)	430 (52.8)
1-3/mo	14 451 (9.9)	10 921 (10.3)	3530 (8.7)	1188 (9.6)	129 (9.1)	81 (10.0)
1-2/wk	6650 (4.6)	5151 (4.9)	1499 (3.7)	502 (4.0)	61 (4.3)	34 (4.2)
≥3/wk	50 143 (34.3)	33 960 (32.1)	16 183 (40.0)	4517 (36.4)	502 (35.2)	269 (33.1)

Abbreviations: CRC, Colorectal Cancer; GI, gastrointestinal.

^a^ Smoking status missing from total cohort (n = 20).

^b^ Body mass index status missing from total cohort (n = 2027).

^c^ Aggregate of Hispanic, Asian, Pacific Islander, and American Indian, owing to low study numbers.

^d^ Calculated as weight in kilograms divided by height in meters squared.

The results of the Cox proportional hazards regression model are presented in Table 2. In our model, any aspirin use was associated with reduced all-cause and cancer-specific mortality. Reduced risk of all-cause mortality was associated with using aspirin 1 to 3 times per month (HR, 0.84; 95% CI, 0.80-0.88; *P* < .001), 1 to 2 times per week (HR, 0.86; 95% CI, 0.81-0.90; *P* < .001), or 3 or more times per week (HR, 0.81; 95% CI, 0.80-0.83; *P* < .001). A similar association was noted for risk of cancer mortality and aspirin use 1 to 3 times per month (HR, 0.87; 95% CI, 0.81-0.94; *P* < .001) or 3 or more times per week (HR, 0.85; 95% CI, 0.81-0.88; *P* < .001). Use of aspirin 3 or more times per week was also associated with significantly reduced risk of GI cancer mortality (HR, 0.75; 95% CI, 0.66-0.84; *P* < .001) and CRC mortality (HR, 0.71; 95% CI, 0.61-0.84; *P* < .001). Data for the additional covariates included in the model are provided in the eTable in the Supplement.

**Table 2.** Updated Aspirin Use and All-Cause, Cancer, GI Cancer, and CRC Mortality Among Individuals Aged 65 Years and Older

Reported Aspirin Use	Mortality, Hazard Ratio (95% CI)^a^			
	All-Cause	Cancer	GI Cancer	CRC
None	1 [Reference]	1 [Reference]	1 [Reference]	1 [Reference]
1-3/mo	0.84 (0.80-0.88)	0.87 (0.81-0.94)	0.84 (0.68-1.05)	0.90 (0.69-1.19)
1-2/wk	0.85 (0.81-0.90)	0.90 (0.82-1.00)	0.90 (0.68-1.19)	0.75 (0.50-1.12)
≥3/wk	0.81 (0.79-0.83)	0.85 (0.81-0.88)	0.75 (0.66-0.84)	0.71 (0.61-0.84)

Abbreviations: CRC, colorectal cancer; GI, gastrointestinal.

^a^ Model is additionally adjusted for sex, race/ethnicity, randomization group, smoking status, ibuprofen use 3 or more times per week, and history of myocardial infarction, stroke, hypertension, and diabetes.

**Figure 2.**
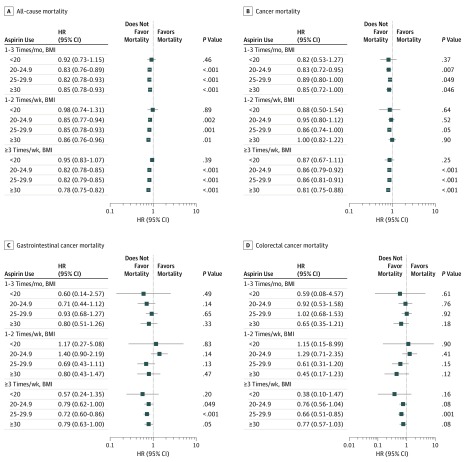
Adjusted Hazard Ratios (HRs) and 95% CIs for Mortality by Aspirin Use Stratified by Body Mass Index (BMI) No aspirin use was used as the reference for all comparisons. The model was adjusted for sex, race/ethnicity, randomization group, smoking status, ibuprofen use 3 times or more per week, and history of myocardial infarction, stroke, hypertension, and diabetes. Body mass index was calculated as weight in kilograms divided by height in meters squared.

To explore the potential association of aspirin use and BMI with mortality risk, we stratified the cohort according to BMI. The results are shown in Figure 2. Compared with no aspirin use and among individuals with a BMI of 20 or higher, aspirin use was associated with reduced risk of all-cause mortality when used 1 to 3 times per month (BMI 20-24.9: HR, 0.83; 95% CI, 0.76-0.89; *P* < .001; BMI 25-29.9: HR, 0.82; 95% CI, 0.78-0.93; *P* < .001; BMI ≥30: HR, 0.85; 95% CI, 0.78-0.93; *P* < .001), 1 to 2 times per week (BMI 20-24.9: HR, 0.85; 95% CI, 0.77-0.94; *P* = .002; BMI 25-29.9: HR, 0.85; 95% CI, 0.78-0.93; *P* = .001; BMI ≥30: HR, 0.86; 95% CI, 0.76-0.96; *P* = .01), or 3 or more times per week (BMI 20-24.9: HR, 0.82; 95% CI, 0.78-0.85; *P* < .001; BMI 25-29.9: HR, 0.82; 95% CI, 0.79-0.85; *P* < .001; BMI ≥30: HR, 0.78; 95% CI, 0.75-0.82; *P* < .001) (Figure 2A). Reduced risk of cancer-specific mortality was noted among individuals with reported aspirin use 3 or more times per month and BMI 20 to 24.9 (HR, 0.86; 95% CI, 0.79-0.92; *P* < .001), BMI 25-29.9 (HR, 0.86; 95% CI, 0.81-0.91; *P* < .001) or BMI 30 or higher (HR, 0.81; 95% CI, 0.75-0.88; *P* < .001) (Figure 2B). No statistically significant risk reduction was observed for cancer mortality among participants who used aspirin 1 to 2 times per week or 1 to 3 times per month, except for aspirin use 1 to 3 times per month among individuals with BMI 20 to 24.9 (HR, 0.83; 95% CI, 0.72-0.95; *P* = .007) (Figure 2B). Individuals with BMI 25 to 29.9 who used aspirin 3 or more times per week were associated with reduced risk of GI cancer mortality (HR, 0.72; 95% CI, 0.60-0.86; *P* < .001) and CRC mortality (HR, 0.66; 95% CI, 0.51-0.85; *P* < .001) (Figure 2C and D).

We additionally examined the association between reported aspirin use at baseline and at the time of the SQ with risk of mortality. Participants who had reported aspirin use at follow-up only or consistent users were associated with a reduced risk of mortality (Table 3). Individuals who reported aspirin use at follow-up only were associated with reduced risk of all-cause mortality (HR, 0.79; 95% CI, 0.76-0.82; *P* < .001), cancer mortality (HR, 0.82; 95% CI, 0.76-0.88; *P* < .001), and GI cancer mortality (HR, 0.74; 95% CI, 0.59-0.94; *P* = .01). Individuals who were consistent aspirin users were also associated with reduced risk of all-cause mortality (HR, 0.80; 95% CI, 0.76-0.83; *P* < .001), cancer mortality (HR, 0.77; 95% CI, 0.72-0.83; *P* < .001), and GI cancer mortality (HR, 0.75; 95% CI, 0.59-0.94; *P* = .01). Of particular interest, the HRs for CRC-associated mortality were 0.88 (95% CI, 0.58-1.33; *P* = .53) for users at baseline only, 0.62 (95% CI, 0.46-0.85; *P* = .003) for users at follow-up only, and 0.62 (95% CI, 0.46-0.85; *P* = .003) for consistent users, compared with nonusers of aspirin.

**Table 3.** Baseline and Follow-up Aspirin Use and Risk of All-Cause, Any Cancer, GI Cancer, and CRC Mortality Among Individuals Aged 65 Years and Older

Reported Aspirin Use	No.	Mortality^a^							
		All-Cause		Cancer		GI Cancer		CRC	
		Deaths	HR (95% CI)	Deaths	HR (95% CI)	Deaths	HR (95% CI)	Deaths	HR (95% CI)
Total	101 098	17 610		5396		559		308	
Consistent nonusers	32 658	5185	1 [Reference]	1771	1 [Reference]	189	1 [Reference]	117	1 [Reference]
Users at baseline	9259	2015	1.06 (1.00-1.12)	570	0.99 (0.90-1.10)	67	1.10 (0.82-1.48)	35	0.88 (0.58-1.33)
Users at follow-up	27 583	4804	0.79 (0.76-0.82)	1497	0.82 (0.76-0.88)	148	0.74 (0.59-0.94)	80	0.62 (0.46-0.85)
Consistent users	28 186	5606	0.80 (0.76-0.83)	1558	0.77 (0.72-0.83)	155	0.75 (0.59-0.94)	76	0.62 (0.46-0.85)

Abbreviations: CRC, colorectal cancer; GI, gastrointestinal; HR, hazard ratio.

^a^ Model is adjusted for sex, race/ethnicity, randomization group, body mass index, ibuprofen use 3 or more times/week, smoking status, and history of myocardial infarction, stroke, hypertension, and diabetes.

## Discussion

This cohort study found that aspirin use among individuals 65 years and older was associated with a lower risk of mortality. This observation was consistent across all causes of mortality (ie, all-cause, cancer, GI cancer, and CRC); however, the greatest reduction in risk was noted for CRC mortality among individuals who used aspirin 3 or more times per week. Additionally, our exploratory analyses investigating the potential associations among aspirin use, BMI, and mortality risk suggest that the efficacy of aspirin as a cancer preventive agent may be associated with BMI. Participants in the PLCO Cancer Screening Trial who were underweight (ie, BMI <20) had no observable benefit associated with aspirin use, while those with BMI 20 or higher were associated with reduced mortality risk, particularly with aspirin use 3 or more times per week. reduced risk of CRC mortality was only associated with individuals with BMI 20 to 29.9 who reported aspirin use 3 or more times per week.

The efficacy of prophylactic aspirin use for prevention of cancer incidence and mortality has been debated; however, the most evidence from prospective cohorts and secondary analyses from clinical trials indicates a protective association with aspirin use. A 2016 systematic analysis of primary and secondary cardiovascular prevention trials^21^ found reduced CRC incidence 10 to 19 years after aspirin use initiation. This association persists among investigations of aspirin use and cancer mortality. A 2011 systematic analysis of 8 clinical trials^22^ found that daily aspirin use was associated with reduced risk of death of several cancers, with increased benefit associated with long-term use. A similar association was observed in a 2018 cohort study^23^ of veterans in which aspirin users were associated with reduced risk of CRC mortality compared with nonusers. This association was additionally examined in the Cancer Prevention Study-II Nutrition Cohort,^24^ in which daily aspirin use was associated with reduced overall cancer mortality. These observations are in contrast with data from the ASPREE trial.^13^ However, the interpretation of the ASPREE results is limited owing a lack of an association of aspirin with cancer and CRC incidence and the short duration of follow-up.^25^ With additional follow-up, an association of aspirin with lower cancer incidence and death may have emerged.^26^ In addition, a 2018 combined analysis of the NIH-AARP Diet and Health Study and the PLCO Cancer Screening Trial^27^ reported decreased risk of all-cause, cancer, and cardiovascular mortality associated with daily aspirin use. However, a dosage that exceeded 1 per day was associated with an increased risk of mortality. These data also did not account for effect modifications by BMI on mortality risk. Previous studies have also found that variables, such as BMI, are associated with the efficacy of prophylactic aspirin. In a 2012 study of the Cancer Prevention Study-II Nutrition Cohort,^28^ individuals with prediagnostic BMI 30 or higher were associated with increased risk of all-cause and CRC death. A similar association was demonstrated across several other cohort and case-control studies, cancers, and causes of death.^27,29,30,31^

The observation that BMI may be associated with efficacy of aspirin in individuals 65 years and older is notable; however, our findings require further confirmation. Increasing rates of overweight and obesity globally may substantially alter the population-based efficacy of cancer prevention prophylatics.^32^ Studies have suggested that aspirin has reduced effectiveness as a primary prevention modality among individuals who are obese owing to decreased bioavailability and antithrombotic efficacy; however, this study did not find an association of overweight or obesity with decreased efficacy.^5,33,34,35^ Therefore, although aspirin use is associated with benefit as a cancer preventive agent, the changing characteristics of the global population may alter its efficacy and must be considered along with age and risk of bleeding before recommending aspirin for cancer prevention.

### Limitations and Strengths

Our study has several limitations. First, this is a secondary analysis of a randomized intervention (screening) trial with self-reported data on aspirin use. When collecting self-reported data, one must be aware of the limitations to these data, such as underreporting parameters of interest and the potential effect of this error in the analyses, such as biasing toward the null. For example, it is possible that aspirin use was underreported if the participant was unaware that a given agent, based upon its trade name, was classified as aspirin. Next, the information on aspirin use may be subject to participant interpretation and measurement error. Regular use of aspirin was not defined in the BQ, so participants could then have interpreted regular aspirin use differently, which may have affected their categorization (eg, none, monthly, weekly, daily). Furthermore, the information collected regarding aspirin dosage was limited. These issues may, in addition to confounding by unmeasured covariates, help explain the lack of a dose-response association for mortality risk in the observed data. Though there is substantial evidence documenting the association of frequent aspirin use with cancer prevention, the biological effect of low frequency use (eg, 1-3 times per month) is uncertain. Therefore, the noted risk reduction associated with this group may be due to unmeasured confounders. Along similar lines, it remains possible that individuals who reported low or no use of aspirin were unable to tolerate aspirin use for an extended length of time, perhaps owing to risk of bleeding or gastrointestinal injury, and may be inherently different than those who reported aspirin use.^36^ Additionally, our analysis was focused on individuals in the PLCO Cancer Screening Trial cohort who survived to and began follow-up at or after age 65 years. However, only a small percentage (5.5%) of the PLCO cohort was ineligible for our study.

Although this study had several limitations, the research questions investigated examining the associations among aspirin use, BMI, and mortality adds to our knowledge of modifiable factors associated with cancer prevention efficacy. We were able to explore this question in a large, prospective study with an extended follow-up period.

## Conclusions

In this cohort study, we found a significant association of aspirin use with reduced all-cause, any cancer, GI cancer, and CRC mortality among individuals 65 years and older in the PLCO Cancer Screening Trial. Aspirin use was associated with reduced risk of all-cause and any cancer mortality, and aspirin use 3 or more times per week was associated with reduced risk of GI cancer mortality and CRC mortality when stratified by BMI. Future studies should further examine the association of BMI with the efficacy of aspirin as a cancer preventive agent to adapt to the changing global obesity trends.

## Notes

Supplement. **eTable.** Covariates Included in the Cox Proportional Regression Hazard Model
