# Error in Results Section of the Abstract

**DOI:** 10.1001/jamanetworkopen.2019.21081

**Published:** 2020-01-17

In the Original Investigation titled “Association of Aspirin Use With Mortality Risk Among Older Adult Participants in the Prostate, Lung, Colorectal, and Ovarian Cancer Screening Trial,”^1^ published December 4, 2019, there was an error in the data given in the Results section of the Abstract. The hazard ratio (HR) and 95% CI for any cancer mortality among individuals with body mass index 20 to 24.9 who used aspirin 3 or more times per week should have read “HR, 0.86; 95% CI, 0.79-0.92.” This article has been corrected.^1^
