# Supplementary material for: Association of Aspirin Use With Mortality Risk Among Older Adult Participants in the Prostate, Lung, Colorectal, and Ovarian Cancer Screening Trial
Source: JAMA Netw Open. 2019 Dec 4;2(12):e1916729. doi: 10.1001/jamanetworkopen.2019.16729 (PMC6902761; doi:10.1001/jamanetworkopen.2019.16729)
Supplement: Supplement. — eTable. Covariates Included in the Cox Proportional Regression Hazard Model [file jamanetwopen-2-e1916729-s001.pdf]

Supplementary Online Content

Loomans-Kropp HA, Pinsky P, Cao Y, Chan AT, Umar A. Association of aspirin use with mortality risk among older adult participants in the Prostate, Lung, Colorectal, and Ovarian Cancer Screening Trial. *JAMA Netw Open*. 2019;2(12):e1916729. doi:10.1001/jamanetworkopen.2019.16729

eTable. Covariates Included in the Cox Proportional Regression Hazard Model

This supplementary material has been provided by the authors to give readers additional information about their work.

© 2019 Loomans-Kropp HA et al. *JAMA Network Open.*

eTable. Covariates Included in the Cox Proportional Regression Hazard Model

	All-cause mortality	Cancer mortality	GI cancer mortality	CRC mortality
Variable	Adjusted HR (95% CI)	Adjusted HR (95% CI)	Adjusted HR (95% CI)	Adjusted HR (95% CI)
Sex				
Male	1.0 (Ref)	1.0 (Ref)	1.0 (Ref)	1.0 (Ref)
Female	0.68 (0.67, 0.70)	0.70 (0.67, 0.73)	0.49 (0.43, 0.55)	0.65 (0.56, 0.76)
Race				
White, non-Hispanic	1.0 (Ref)	1.0 (Ref)	1.0 (Ref)	1.0 (Ref)
Black, non-Hispanic	1.22 (1.17, 1.27)	1.19 (1.11, 1.29)	1.65 (1.34, 2.03)	1.72 (1.32, 2.25)
Other	0.81 (0.78, 0.85)	0.82 (0.76, 0.89)	1.14 (0.93, 1.39)	1.02 (0.78, 1.35)
Randomization arm				
Control	1.0 (Ref)	1.0 (Ref)	1.0 (Ref)	1.0 (Ref)
Intervention	1.02 (1.00, 1.05)	1.02 (0.98, 1.05)	1.25 (1.12, 1.39)	1.34 (1.16, 1.54)
Smoking status				
Never smoker	1.0 (Ref)	1.0 (Ref)	1.0 (Ref)	1.0 (Ref)
Current smoker	2.76 (2.68, 2.85)	3.68 (3.48, 3.89)	1.95 (1.62, 2.34)	1.45 (1.12, 1.87)
Former smoker	1.31 (1.28, 1.34)	1.58 (1.52, 1.65)	1.35 (1.20, 1.52)	1..20 (1.03, 1.40)
BMI				
<20	1.43 (1.36, 1.50)	1.18 (1.07, 1.30)	1.36 (1.01, 1.82)	1.03 (0.69, 1.54)
20-24.9	1.0 (Ref)	1.0 (Ref)	1.0 (Ref)	1.0 (Ref)
25-29.9	0.88 (0.86, 0.90)	0.98 (0.94, 1.02)	0.98 (0.86, 1.12)	0.99 (0.83, 1.18)
≥30	1.03 (1.00, 1.06)	1.09 (1.04, 1.15)	1.19 (1.02, 1.39)	1.27 (1.04, 1.55)
History of heart attack				
No				
Yes	1.0 (Ref)	1.0 (Ref)	1.0 (Ref)	1.0 (Ref)
	1.88 (1.83, 1.93)	1.30 (1.23, 1.38)	1.44 (1.23, 1.70)	1.42 (1.14, 1.78)
History of stroke				
No	1.0 (Ref)	1.0 (Ref)	1.0 (Ref)	1.0 (Ref)
Yes	1.57 (1.51, 1.63)	1.11 (1.02, 1.21)	1.16 (0.90, 1.49)	1.18 (0.84, 1.66)
History of hypertension				
No				
Yes	1.0 (Ref)	1.0 (Ref)	1.0 (Ref)	1.0 (Ref)
	1.23 (1.21, 1.26)	1.07 (1.03, 1.11)	1.01 (0.90, 1.13)	0.86 (0.74, 1.00)
History of diabetes				
No	1.0 (Ref)	1.0 (Ref)	1.0 (Ref)	1.0 (Ref)
Yes	1.64 (1.60, 1.69)	1.24 (1.18, 1.31)	1.40 (1.20, 1.64)	1.48 (1.21, 1.82)
Ibuprofen use				
<3 times per week	1.0 (Ref)	1.0 (Ref)	1.0 (Ref)	1.0 (Ref)
≥3 times per week	1.16 (1.12, 1.20)	1.02 (0.96, 1.08)	0.98 (0.81, 1.18)	0.90 (0.69, 1.15)

© 2019 Loomans-Kropp HA et al. *JAMA Network Open.*
